# Global, regional, and national mortality of tuberculosis attributable to alcohol and tobacco from 1990 to 2019: A modelling study based on the Global Burden of Disease study 2019

**DOI:** 10.7189/jogh.14.04023

**Published:** 2024-01-05

**Authors:** Wu Jinyi, Yue Zhang, Kai Wang, Peng Peng

**Affiliations:** 1 Wuhan Fourth Hospital, Wuhan, China; 2 Shanxi Medical University, Taiyuan, China

## Abstract

**Background:**

Tuberculosis (TB) is expected to become the second leading single cause of death with several risk factors, but the related disease burden is currently unknown. We aimed to analyse the pre-coronavirus disease 2019 (COVID-19) changes in mortality of TB attributable to alcohol and tobacco worldwide from 1990 to 2019.

**Methods:**

We obtained data of TB deaths and age-standardised death rates attributed to alcohol and cigarette in 204 countries and territories from the Global Burden of Disease 2019 public database. We performed a spatial-temporal analysis of age-standardised death rate and the average annual per cent change (AAPC), after which we analysed the effects of gender and socio-demographic index on age-standardised death rate using an age-period-cohort model. Finally, we built machine learning models to predict the TB age-standardised death rate in 2035.

**Results:**

We found that the global age-standardised death rate of TB attributable to alcohol consumption declined from 5.35 (95% uncertainty interval (UI) = 3.51, 7.00) in 1990 to 2.54 (95% UI = 1.65, 3.33) in 2019, with significant declines in Andean Latin America (AAPC = −7.59; 95% confidence interval (CI) = −8, −7.16, *P* < 0.05), East Asia (AAPC = −7.32; 95% CI = −8.00, −6.62, *P* < 0.05), and Central Latin America (AAPC = −7.31; 95% CI = −7.63, −6.99, *P* < 0.05). However, there was an increase in a few regions, especially in parts of Central Asia. The age-standardised death rate for TB attributable to cigarette smoking declined more rapidly than that for TB attributable to alcohol, from 7.45 (95% UI = 6.17, 8.72) to 2.21 (95% UI = 1.78, 2.64), especially in East Asia (AAPC = −6.64; 95% CI = −7.07, −6.2, *P* < 0.05), North Africa and Middle East (AAPC = −6.47; 95% CI = −6.67, −6.28, *P* < 0.05), and Andean Latin America (AAPC = −6.31; 95% CI = −6.87, −5.75, *P* < 0.05). However, TB attributable to cigarette smoking increased in parts of Eastern Europe. In both TB attributable to alcohol consumption and to cigarette smoking, the age-standardised death rate was much higher in men than in women. The age-period-cohort model results showed that TB attributable to alcohol consumption was the highest in older adults aged 60-80 years, while TB attributable to cigarette smoking was the highest in adults aged 40-60 years. Machine learning models projected that by 2035, the age-standardised death rate for TB attributable to alcohol consumption would be 1.29 (per 100 000 population), while the age-standardised death rate for TB attributable to cigarette consumption would be 0.37 (per 100 000 population), which might not achieve the 2035 global target for eliminating TB.

**Conclusions:**

Globally, the age-standardised death rate for TB attributable to alcohol consumption declined slower than that attributable to cigarette smoking. Controlling these two factors would help achieve the global goal of TB elimination, especially for the elderly who are at high risk.

Tuberculosis (TB) is a single infectious agent disease caused by mycobacterium tuberculosis (Mtb) and is the 13th leading cause of death worldwide, usually affecting the lungs and other organs [1]. It is the second leading single cause of death in the future, with an estimated 10 million TB patients globally in 2020 and 10.6 million in 2021. It is reported that 1.5 million in 2020 and 1.6 million in 2021 will die from TB [2]. In response, the United Nations Sustainable Development Goals (SDG) and the World Health Organization (WHO) set the targets of eliminating TB by reducing mortality by 95% and incidence by 90% by 2035 [3,4]. Meanwhile, global TB incidence declined by about 2% per year from 2015 to 2020, far below the SDG targets, with the cumulative decline (11%) being only half of the strategic milestone of 20% reduction [2]. The coronavirus disease 2019 (COVID-19) pandemic might have further affected this, leading to increases in TB deaths and stagnation in elimination efforts [5].

The new TB cases were attributed to five main risk factors: malnutrition, human immunodeficiency virus (HIV) infection, alcohol disorders, cigarettes (especially in men), and diabetes [6]. Among these, the tobacco epidemic, which kills more than eight million people each year, remains a serious global public health threat. According to recent reports, the global smoking prevalence remains high at 20%, yet varies widely between countries, from 8.7% in Sweden to 27% in Greece and Bulgaria [7]. Meanwhile, epidemiologic evidence has shown that tobacco smoke exposure is associated with increased TB infection, while its adverse impacts on the transmissibility of TB are reduced shortly after an individual quits smoking, reinforcing the importance of smoking cessation interventions in TB control [8]. According to the WHO Global Trends in Cigarette Epidemic 2021 report, there was an estimated 17.6% of TB cases and 15.2% of TB deaths attributable to cigarette smoking in 2021.

Meanwhile, an estimated 107 million people suffer from alcohol use disorder globally, resulting in 2.8 million premature deaths each year [9]. Previous studies have shown that alcohol consumption is associated with symptom severity and death in TB patients [10], while the Global Trends in Alcohol Epidemic 2018 report estimated that 19.6% of TB prevalence was attributable to alcohol use [11].

As global health goals emphasise the importance of tobacco and alcohol consumption management for TB prevention and control, understanding trends of TB age-standardised death rates (ASDRs) is key for progressing toward the 2035 TB control targets and informing related policies and programmes. In response, we aimed to estimate the pre-COVID-19 trend of TB mortality attributed to alcohol and tobacco from 1990 to 2019.

## METHODS

### Data sources

We obtained data from the Global Burden of Disease (GBD) 2019, which covered 369 diseases and injuries with 87 risk factors in 204 countries and territories [12,13], and whose methods and lethality and non-lethality estimates have been published elsewhere [14,15].

All countries and territories were grouped into 21 regions (based on epidemiological similarity and geographical proximity) and into five categories based on socio-demographic index (SDI) (high SDI>0.81, high-medium SDI = 0.70–0.81, medium SDI = 0.61–0.69, medium-low SDI = 0.46–0.60, and low SDI<0.46). The SDI is a composite indicator of the social context and economic conditions that affect the health of each country and region [16], calculated as a geometric mean from 0 to 1 and comprising per capita income, the average educational attainment of the population aged 15 years and older, and the fertility rate of women under 25 years.

We restricted our analysis indicator to age-specific death rates (ASDRs) of TB, diagnosed in the GBD 2019 per the International Classification of Diseases, 10th Revision (ICD-10). This data had the same limitations as the general GBD data, such as the fact that the disease surveillance site system did not cover some remote and poor areas and that data were derived from a highly informed GBD model in a high-resource setting.

### Statistical analysis

#### Descriptive analysis

**Figure 1:**
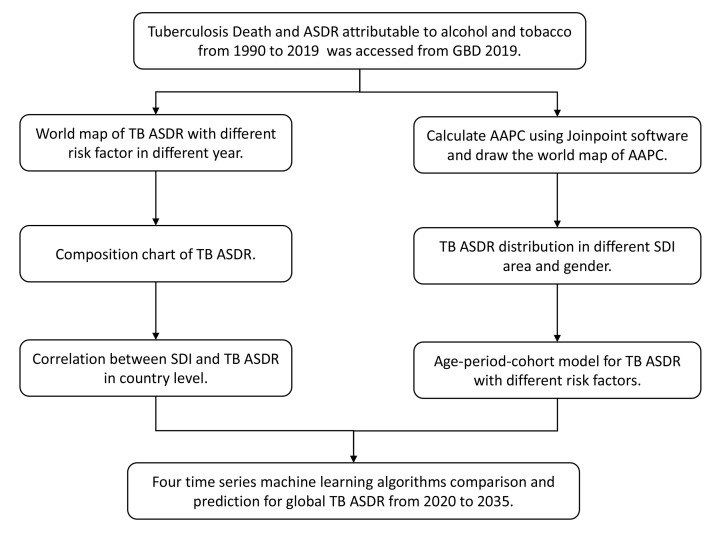
Flowchart for the design of this research.

We screened for risk factors of TB and selected cigarette smoking and alcohol consumption as research topics, since they were two main risk factors of TB. We drew a world map of 204 countries to visualise the ASDR and average annual percent change (AAPC) of TB attributable to cigarette (TBC) with TB attributable to alcohol (TBA) in 1990 and 2019. We then calculated TBC with TBA mortality and their changes for different genders in 21 GBD regions and assessed the relationship between ASDR and SDI in different countries to explore potential social factors influencing ASDR (**Figure 1**). All AAPC [5] calculations were performed using Joinpoint software, version 4.9.1.0. (National Cancer Institute, Bethesda, Maryland, USA), while all other analyses were conducted in R, version 3.5.2. (R Core Team, Vienna, Austria). We considered *P* < 0.05 as statistically significant.

#### Age-period-cohort model

The APC model addressed the linear relationship between age, period of research. and birth cohort of samples by using an intrinsic estimation method for separating their relative effects [17]:

*Y*  = log(*M*) = μ + *αX*1 + *βX*2 + *γX*3 + ϵ

Here, *M* was the death rate, while α, β, and γ were the effect coefficients for age, period, and cohort, with μ and ε as the intercept and random error, respectively. We divided ASDR into 14 age groups (25–29, 30–34, 35–39, 40–44, 45–49, 50–54, 55–59, 60–64, 65–69, 70–74, 75–79, 80–84, 85–89, and >90 years), five consecutive study periods (1994–98, 1999–2003, 2004–08, 2009–13, 2014–19), and consecutive five–year birth cohort groups (1904–08, 1909–13, 1914–18, 1919–23, 1924–28, 1929–33, 1934–38, 1939–43, 1944–48, 1949–53, 1954–58, 1959–63, 1964–68, 1969–73, 1974–78, 1979–83, 1984–88, and 1989–94) [17].

We used an APC model to estimate the independent effects of age, period, and birth cohort on TB ASDR, in which the net drift represented the log-linear trend in period and cohort for the entire population, and the local drift represented the log-linear trend in period and cohort for each age group. Model results showed longitudinal age profiles, period risk, and cohort risk.

#### Machine learning prediction for time series

Several types of models among machine learning (ML) algorithms were available for time series prediction [18,19], where temporal information could be included by adding to the input a set of delays to represent the data at different time points [20-22]. We the “modeltime” package in R software for four ML algorithms (autoregressive integrated moving average (ARIMA), Prophet, Elastic Net, and XGBoost) to predict ASDR of TBC and TBA until 2035. The ARIMA statistical analysis model employs time-series data to help researchers better understand a data collection or to forecast future trends. Meanwhile, a Prophet model is a Facebook open-source platform for forecasting time series data that lets companies better interpret and anticipate demand. Elastic Net is a particular case of the shrinkage method, which holds both ridge and least absolute shrinkage and selection operator (LASSO) regressions. The XGBoost model is an ensemble ML system for tree boosting based on the gradient direction of a loss function called gradient boosting machine [23].

### Ethics approval

We did not require ethical approval for our study specifically, as the GBD data were unidentified and aggregated by the University of Washington Institute for Health Metrics and Evaluation, which received informed consent waivers approval from the University of Washington Institutional Review Board.

## RESULTS

**Figure 2:**
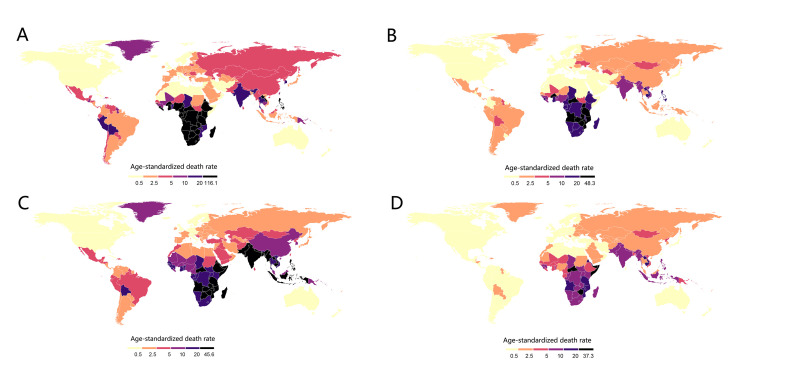
**Panel A.** Alcohol-related ASDR 1990. **Panel B.** Alcohol-related ASDR 2019. **Panel C.** Tobacco-related ASDR 1990. **Panel D.** Tobacco-related ASDR 2019. ASDR – age-standardised death rate.

In our analysis of 204 countries, TBA ASDR declined in globally from 5.35 (95% uncertainty interval (UI) = 3.51, 7.00) in 1990 to 2.54 (95% UI = 1.65, 3.33) in 2019, but primarily in East Asia (AAPC = −7.32; 95% confidence interval (CI) = −8.00, −6.62, *P* < 0.05) and its sub-regions of China and Russia, Andean Latin America (AAPC = −7.59; 95% CI = −8.00, −7.16, *P* < 0.05), and Central Latin America (AAPC = −7.31; 95% CI = 7.63, −6.99, *P* < 0.05) (**Figure 2**, Panels A-B). TBC ASDR declined more rapidly worlwide, from 7.45 (95% UI = 6.17, 8.72) to 2.21 (95% UI = 1.78, 2.64), particularly in East Asia (AAPC = −6.64; 95% CI = −7.07, −6.20, *P* < 0.05), North Africa and Middle East (AAPC = −6.47; 95% CI = −6.67, −6.28, *P* < 0.05), and Andean Latin America (AAPC = −6.31; 95% CI = −6.87, −5.75, *P* < 0.05). (**Figure 2**, Panels C-D).

**Figure 3:**
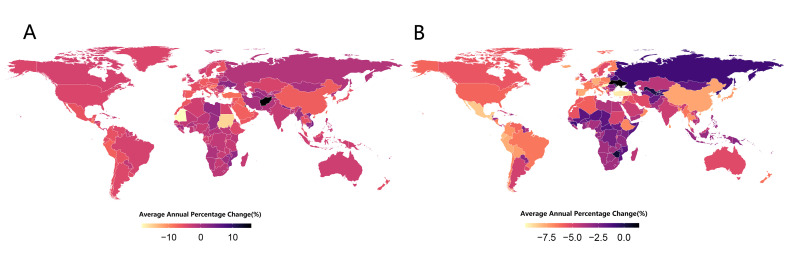
**Panel A.** Alcohol-related AAPC 1990. **Panel B.** Tobacco-related AAPC 2019. AAPC – average annual percent change.

According to the AAPC for each country, TBA ASDR decreased in most regions worldwide from 1990 to 2019, yet increased in a small number of regions, especially in East Asia (AAPC = −6.64; 95% CI = −7.07, −6.20, *P* < 0.05), North Africa and Middle East (AAPC = −6.47; 95% CI = −6.67, −6.28, *P* < 0.05), and Andean Latin America (AAPC = −6.31; 95% CI = −6.87, −5.75, *P* < 0.05). Meanwhile, TBC ASDR decreased worldwide (**Table 1** and **Figure 3**, Panels A-B)

**Table 1:** Global burden of TB attributable to alcohol and tobacco in 1990 and 2019, and the temporal trends from 1990 to 2019

	1990		2019		
**Characteristics**	**Death, n ×104 (95% UI)**	**ASDR per 100 000, n (95% UI)**	**Death, n ×104 (95% UI)**	**ASDR per 100 000, n (95% UI)**	**AAPC for ASDR (1990–2019), n (95% UI)**
**Attributable to cigarette smoking**					
Overall	30.94 (25.65, 36.23)	7.45 (6.17, 8.72)	18.31 (14.79, 21.9)	2.21 (1.78, 2.64)	−4.07 (−4.19, −3.95)
Sex					
*Males*	28.28 (23.47, 33.14)	14.6 (12.1, 17.09)	16.8 (13.57, 20.18)	4.24 (3.43, 5.09)	−4.13 (−4.26, −4.01)
*Females*	2.67 (1.93, 3.49)	1.23 (0.89, 1.61)	1.51 (1.09, 1.98)	0.35 (0.25, 0.46)	−4.26 (−4.48, −4.04)
SDI region					
*High SDI*	0.86 (0.734, 0.997)	0.84 (0.72, 0.97)	0.25 (0.201, 0.293)	0.13 (0.11, 0.16)	−5.31 (−5.50, −5.12)
*High-middle SDI*	3.16 (2.71, 3.66)	2.86 (2.45, 3.31)	1.33 (1.13, 1.56)	0.68 (0.57, 0.79)	−3.97 (−4.69, −3.25)
*Low SDI*	4.72 (3.55, 5.92)	18.33 (13.63, 23.09)	3.63 (2.7, 4.58)	6.25 (4.65, 7.83)	−2.59 (−2.77, −2.41)
*Low-middle SDI*	13.34 (10.94, 15.86)	21.15 (17.17, 25.17)	8.23 (6.52, 10.17)	5.78 (4.57, 7.15)	−2.12 (−2.60, −1.63)
*Middle SDI*	8.84 (7.32, 10.4)	8.31 (6.86, 9.87)	4.85 (3.97, 5.76)	1.92 (1.57, 2.28)	−3.46 (−3.67, −3.25)
GBD region					
*Andean Latin America*	0.11 (0.09, 0.14)	5 (3.79, 6.22)	0.03 (0.02, 0.04)	0.51 (0.34, 0.7)	−6.31 (−6.87, −5.75)
*Australasia*	0.003 (0.003, 0.004)	0.14 (0.11, 0.16)	0.001 (0.001, 0.002)	0.03 (0.02, 0.04)	−3.69 (−4.14, −3.25)
*Caribbean*	0.03 (0.03, 0.04)	1.28 (1, 1.57)	0.02 (0.02, 0.03)	0.44 (0.31, 0.58)	−2.7 (−3.14, −2.26)
*Central Asia*	0.15 (0.13, 0.17)	2.86 (2.48, 3.26)	0.14 (0.12, 0.17)	1.54 (1.28, 1.82)	−1.6 (−2.16, −1.04)
*Central Europe*	0.23 (0.2, 0.26)	1.6 (1.4, 1.82)	0.06 (0.05, 0.08)	0.36 (0.3, 0.44)	−4.28 (−4.68, −3.89)
*Central Latin America*	0.21 (0.17, 0.25)	2.38 (1.89, 2.82)	0.06 (0.05, 0.08)	0.27 (0.19, 0.34)	−5.55 (−5.89, −5.21)
*Central sub-Saharan Africa*	0.48 (0.33, 0.65)	17.72 (12.12, 23.73)	0.56 (0.37, 0.8)	8.2 (5.44, 11.6)	−1.79 (−2.01, −1.57)
*East Asia*	4.92 (3.98, 6.00)	5.44 (4.39, 6.63)	1.28 (0.99, 1.60)	0.62 (0.48, 0.77)	−6.64 (−7.07, −6.2)
*Eastern Europe*	0.55 (0.48, 0.62)	2 (1.75, 2.25)	0.48 (0.4, 0.58)	1.66 (1.37, 2.01)	−0.1 (−1.94, 1.76)
*Eastern sub-Saharan Africa*	1.95 (1.40, 2.50)	23.55 (16.83, 30.37)	1.61 (1.11, 2.21)	8.25 (5.61, 11.35)	−2.96 (−3.07, −2.86)
*High-income Asia Pacific*	0.45 (0.38, 0.51)	2.22 (1.91, 2.55)	0.12 (0.1, 0.15)	0.26 (0.21, 0.3)	−5.9 (−6.24, −5.56)
*High-income North America*	0.08 (0.06, 0.09)	0.23 (0.18, 0.27)	0.02 (0.02, 0.03)	0.04 (0.03, 0.05)	−4.3 (−4.41, −4.20)
*North Africa and Middle East*	0.45 (0.36, 0.56)	2.47 (1.94, 3.05)	0.22 (0.17, 0.28)	0.47 (0.36, 0.60)	−6.47 (−6.67, −6.28)
*Oceania*	0.03 (0.02, 0.04)	8.17 (5.25, 11.67)	0.03 (0.02, 0.05)	3.71 (2.36, 5.33)	−2.99 (−3.1, −2.87)
*South Asia*	13.97 (11.17, 16.85)	23.61 (18.72, 28.62)	8.48 (6.58, 10.68)	5.85 (4.55, 7.34)	−2.45 (−3.35, −1.54)
*Southeast Asia*	5.34 (4.32, 6.43)	20.31 (16.3, 24.65)	3.6 (2.9, 4.38)	5.84 (4.71, 7.13)	−1.6 (−1.81, −1.39)
*Southern Latin America*	0.07 (0.06, 0.08)	1.54 (1.3, 1.77)	0.02 (0.02, 0.03)	0.31 (0.25, 0.37)	−4.87 (−5.05, −4.69)
*Southern sub-Saharan Africa*	0.6 (0.47, 0.72)	18.8 (14.84, 22.84)	0.62 (0.47, 0.76)	9.49 (7.27, 11.65)	−1.23 (−1.84, −0.62)
*Tropical Latin America*	0.27 (0.23, 0.31)	2.58 (2.17, 2.96)	0.1 (0.08, 0.12)	0.38 (0.31, 0.46)	−3.75 (−3.89, −3.6)
*Western Europe*	0.26 (0.22, 0.31)	0.47 (0.4, 0.55)	0.06 (0.05, 0.07)	0.07 (0.06, 0.08)	−5.44 (−5.78, −5.1)
*Western sub-Saharan Africa*	0.79 (0.58, 1.02)	8.19 (5.93, 10.51)	0.78 (0.57, 1.02)	3.53 (2.59, 4.59)	−2.16 (−2.36, −1.95)
**Attributable to alcohol consumption**					
Overall	22.91 (15.07, 29.96)	5.35 (3.51, 7)	20.96 (13.64, 27.42)	2.54 (1.65, 3.33)	−2.52 (−2.67, −2.36)
Sex					
*Males*	20.21 (13.4, 26.22)	10 (6.6, 12.92)	18.53 (12.15, 24.18)	4.69 (3.06, 6.12)	−2.57 (−2.74, −2.4)
*Females*	2.7 (1.60, 3.90)	1.21 (0.72, 1.76)	2.42 (1.41, 3.55)	0.57 (0.33, 0.84)	−2.59 (−2.74, −2.44)
SDI region					
*High SDI*	1.04 (0.75, 1.27)	1.02 (0.74, 1.25)	0.42 (0.29, 0.54)	0.21 (0.14, 0.27)	−6.12 (−6.27, −5.97)
*High-middle SDI*	2.95 (2.06, 3.68)	2.66 (1.85, 3.33)	1.58 (1.08, 2.01)	0.82 (0.57, 1.05)	−4.87 (−5.42, −4.31)
*Low SDI*	5.55 (3.44, 7.55)	20.2 (12.42, 27.45)	5.75 (3.54, 7.88)	9.46 (5.81, 12.97)	−3.64 (−3.90, −3.38)
*Low-middle SDI*	7.06 (4.37, 9.73)	10.3 (6.36, 14.26)	8.19 (5.24, 10.95)	5.49 (3.49, 7.39)	−4.4 (−4.83, −3.97)
*Middle SDI*	6.31 (4.20, 8.24)	5.54 (3.66, 7.27)	5 (3.26, 6.50)	1.99 (1.30, 2.60)	−4.91 (−5.11, −4.71)
GBD region					
*Andean Latin America*	0.31 (0.20, 0.41)	11.53 (7.25, 15.42)	0.1 (0.07, 0.15)	1.71 (1.08, 2.50)	−7.59 (−8.00, −7.16)
*Australasia*	0.01 (0.001, 0.01)	0.25 (0.19, 0.31)	0 (0.001, 0.01)	0.09 (0.06, 0.12)	−5.52 (−5.94, −5.09)
*Caribbean*	0.08 (0.05, 0.1)	2.58 (1.76, 3.38)	0.06 (0.04, 0.08)	1.16 (0.75, 1.59)	−3.57 (−4.09, −3.05)
*Central Asia*	0.16 (0.11, 0.2)	2.91 (1.99, 3.68)	0.17 (0.11, 0.22)	1.77 (1.19, 2.3)	−2.12 (−2.36, −1.88)
*Central Europe*	0.26 (0.19, 0.31)	1.82 (1.35, 2.18)	0.09 (0.07, 0.12)	0.52 (0.37, 0.65)	−5.07 (−5.41, −4.72)
*Central Latin America*	0.3 (0.21, 0.38)	3.04 (2.08, 3.85)	0.14 (0.09, 0.19)	0.58 (0.38, 0.78)	−7.31 (−7.63, −6.99)
*Central sub-Saharan Africa*	0.86 (0.48, 1.31)	31.76 (17.78, 48.49)	1.24 (0.69, 1.92)	18.67 (10.41, 28.91)	−2.6 (−2.80, −2.41)
*East Asia*	3.93 (2.59, 5.19)	4.29 (2.83, 5.67)	1.19 (0.79, 1.59)	0.59 (0.39, 0.79)	−7.32 (−8.00, −6.62)
*Eastern Europe*	0.63 (0.46, 0.75)	2.32 (1.7, 2.79)	0.59 (0.41, 0.74)	2.11 (1.49, 2.65)	−0.60 (−1.69, 0.50)
*Eastern sub-Saharan Africa*	3.47 (2.16, 4.72)	39.47 (24.12, 54.06)	3.32 (2.02, 4.56)	16.58 (10.19, 22.72)	−3.56 (−3.63, −3.49)
*High-income Asia Pacific*	0.51 (0.36, 0.62)	2.57 (1.81, 3.18)	0.22 (0.15, 0.3)	0.44 (0.3, 0.58)	−7.18 (−7.43, −6.93)
*High-income North America*	0.08 (0.05, 0.10)	0.23 (0.16, 0.28)	0.04 (0.03, 0.05)	0.06 (0.04, 0.08)	−5.95 (−6.05, −5.84)
*North Africa and Middle East*	0.11 (0.06, 0.16)	0.54 (0.3, 0.77)	0.04 (0.02, 0.06)	0.08 (0.04, 0.12)	−5.52 (−5.67, −5.37)
*Oceania*	0.02 (0.01, 0.03)	4.93 (2.46, 8.27)	0.02 (0.01, 0.03)	2.05 (0.89, 3.53)	−2.69 (−2.81, −2.58)
*South Asia*	6.87 (4.1, 9.8)	10.29 (6.04, 14.77)	7.62 (4.71, 10.83)	4.99 (3.07, 7.16)	−4.76 (−5.31, −4.20)
*Southeast Asia*	1.88 (1.14, 2.68)	6.53 (3.9, 9.43)	2.5 (1.63, 3.34)	4.05 (2.64, 5.44)	−4.22 (−4.42, −4.01)
*Southern Latin America*	0.12 (0.09, 0.15)	2.64 (1.94, 3.17)	0.05 (0.04, 0.06)	0.62 (0.44, 0.78)	−5.35 (−5.64, −5.05)
*Southern sub-Saharan Africa*	0.77 (0.52, 0.99)	22.51 (15.2, 29.33)	1.04 (0.71, 1.33)	15.67 (10.51, 20.01)	−2.33 (−2.88, −1.78)
*Tropical Latin America*	0.24 (0.16, 0.31)	2.13 (1.38, 2.70)	0.17 (0.12, 0.22)	0.7 (0.48, 0.88)	−6.35 (−6.49, −6.21)
*Western Europe*	0.42 (0.31, 0.5)	0.74 (0.55, 0.88)	0.14 (0.1, 0.18)	0.15 (0.1, 0.18)	−6.49 (−6.83, −6.14)
*Western sub-Saharan Africa*	1.9 (1.16, 2.68)	19.98 (12.1, 28.35)	2.2 (1.39, 2.98)	10.49 (6.56, 14.29)	−2.81 (−3.03, −2.6)

GBD – Global Burden of Disease, SDI – socio-demographic index, UI – uncertainty interval

We performed a cause-of-death structural analysis to understand the composition of TB deaths. Globally, TBA ASDR was lower than that of TBC in 1990, yet this was reversed in 2019. In terms of SDI, TBA was higher than TBC in both high SDI and low SDI regions from 1990 to 2019, while TBA ASDR in high SDI, medium-high SDI, medium SDI, and low SDI areas was higher than TBC ASDR in 2019, meaning in all but low and medium SDI areas. This change in the composition of the TB ASDR suggests that the proportion of TBA in the total TB ASDR increased between 1990 and 2019 (Figure S1 in the **Online Supplementary Document**).

We performed further sub-analyses of ASDR and years of life lost (YLLs) from the perspective of gender and SDI regions (**Figure 5**) and found that TBA ASDR was highest among men in low SDI regions, followed by men in medium and low SDI regions, and then women in low SDI regions. Conversely, men in low, low and medium, and medium SDI areas had the highest TBC ASDR (Figure S2 in the **Online Supplementary Document**).

In the analysis of national ASDR in different SDI regions, the TBA ASDR showed an inverted U-shape, being highest in the low and medium SDI regions and low in the low SDI and other SDI regions. TBA ASDR was highest in Central African Republic, Burundi and Lesotho. The TBC ASDR gradually decreased with increasing SDI, with obviously higher ASDR in Somalia from the low SDI region, Central African Republic from the low to medium SDI region Lesotho, Kiribati, and Zimbabwe from the medium SDI region (Figure S3 in the **Online Supplementary Document**).

The APC model showed an inverted U-shaped ASDR for TBA, with the highest ASDR in the 60-80 age group. Meanwhile, the highest age range of ASDR for TBC was between 40-60 years. In the research of period analysis, the ASDR for both TBA and TBC decreased gradually over time. The 1900−20 birth cohort had higher TBA ASDR than those born in other periods, while 1920–40 birth cohort had higher TBC ASDR than others (Figure S4 in the **Online Supplementary Document**).

To predict the future trend of TB ASDR, we trained four time-series ML models based on ASDR data from 1990–2019, which we first sliced in an 8:2 ratio, meaning we assigned 80% as the training data and 20% as the prediction data. We used low root mean square error (RMSE) as the criterion for a well-trained model. After training, we returned all into the model to make predictions for 2020-2035 to see if the WHO target of eliminating TB by 2035 could be reached. As seen from the inter-model evaluation metrics including mean square error, mean absolute percentage error, mean absolute scaled error, symmetric mean absolute percentage error, RMSE and R2 (**Table 2**), the XGboost model had the best prediction results. The results for both TBC and TBA forecasting showed that ASDR would continue the downward trend from 1990−2019 (**Table 3** and Figure S5, Panels A-B in the **Online Supplementary Document**).

**Table 2:** Evaluation of four ML models for TB ASDR prediction

Accuracy table	Mean square error	Mean absolute percentage error	Mean absolute scaled error	Symmetric mean absolute percentage error	Root mean square error	R^2^
Alcohol						
*ARIMA (1,1,0) (0,0,1)*	0.08	2.87	0.92	2.92	0.08	1
*PROPHET*	0.11	3.89	1.25	3.97	0.11	1
*Elastic Net (GLMNET)*	0.15	5.45	1.76	5.61	0.15	1
*PROPHET-Xgboost*	0.08	2.78	0.89	2.82	0.08	1
Cigarette						
*ARIMA (1,1,0) (0,0,1)*	0.28	11.88	2.4	12.89	0.32	1
*PROPHET*	0.24	9.94	2.02	10.56	0.26	1
*Elastic Net (GLMNET)*	0.25	10.29	2.12	10.92	0.26	1
*PROPHET-Xgboost*	0.21	8.64	1.75	9.12	0.22	1

**Table 3:** Prediction details of TB attributable to alcohol and cigarette from 2020 to 2035 by XGBoost

Date	2020	2021	2022	2023	2024	2025	2026	2027	2028	2029	2030	2031	2032	2033	2034	2035
Alcohol																
*Value (95% CI)*	2.49 (2.33, 2.65)	2.4 (2.24, 2.55)	2.32 (2.16, 2.48)	2.24 (2.08, 2.4)	2.17 (2.01, 2.33)	2.1 (1.94, 2.25)	2 (1.84, 2.16)	1.92 (1.76, 2.08)	1.85 (1.69, 2.01)	1.77 (1.61, 1.93)	1.68 (1.53, 1.84)	1.62 (1.46, 1.78)	1.54 (1.38, 1.69)	1.46 (1.3, 1.61)	1.38 (1.22, 1.53)	1.29 (1.14, 1.45)
Cigarette																
*Value (95% CI)*	2.12 (1.66, 2.58)	1.99 (1.53, 2.45)	1.87 (1.41, 2.33)	1.75 (1.29, 2.21)	1.64 (1.18, 2.1)	1.54 (1.08, 2)	1.41 (0.95, 1.87)	1.29 (0.83, 1.75)	1.18 (0.72, 1.64)	1.07 (0.61, 1.53)	0.94 (0.48, 1.4)	0.84 (0.38, 1.3)	0.72 (0.26, 1.18)	0.61 (0.15, 0.94)	0.48 (0.02, 0.94)	0.37 (−0.09, 0.83)

CI – confidence interval

## DISCUSSION

Although WHO conducted annual trend analyses of tuberculosis, trends in TBC and TBA ASDR had not been studied. We conducted such assessments based on GBD 2019 data, focusing on reporting pre-COVID-19 TB ASDR from 1990 to 2019 in 204 countries and territories where cigarette and alcohol were the leading causes of death.

Alcohol disorders were reported to be one of the most common TB risk factors globally, only second to malnutrition and higher than HIV and cigarette [2]. We found that ASDR for TBA from 1990–2019 was decreasing worldwide, with apparent decreases in East Asia (especially China and Russia), Andean Latin America, and Central Latin America, yet increases in a smaller number of regions, especially in Central Asia.

When stratified by gender and SDI, the ASDR for TBA was highest among men in low-SDI regions, followed by men in low and medium-SDI regions and women in low-SDI regions, meaning that men had higher ASDR than women, while low SDI areas had higher ASDR than medium and high SDI areas. This was possibly related to alcohol consumption, as men generally drink more alcohol and more frequently than women, while the drinking population and drinking habits are higher in low SDI areas than in medium and high SDI areas.

The finding that ASDR of TB was apparently higher in men than in women is consistent with previous studies. The prevalence of bacteriologically confirmed TB was 2.21 times higher in men than in women, and 50−70% higher in men than in women in selected low- and middle-income countries. Evidence suggests that because men have lower levels of health care use, more advanced disease at the time of seeking treatment, and poorer compliance with anti-TB treatment [24].

The results of correlation between SDI and ASDR showed that the TBA ASDR had an inverted U-shape, which might be because very low SDI areas would have a smaller drinking population than low and medium SDI areas due to their low economic level and low ability to purchase alcohol. The APC model showed that the highest wave of TBA ASDR was at the age of 60–80 years, while cohorts born in 1900−20 had apparently higher TBA ASDR than others, indicating a high risk of death in the older drinking TB population. Research has suggested that the combination of later TB treatment initiation and poorer treatment adherence might contribute to a higher hazard of TB at older than in younger age groups [25,26].

The results of the analysis of TBC differed from that of TBA, where TBC ASDR declined more rapidly worldwide, especially in East Asia, North Africa and Middle East, and Andean Latin America, a decline further confirmed by our AAPC findings. In the sub-analyses, men in low, low to moderate SDI areas, moderate SDI areas had the highest TBC ASDR, suggesting it decreased progressively with increasing SDI, in line with previous studies [26,27]. The proportion of TBA and TBC ASDR was more than four and six times higher in men than in women, respectively [27,28]. Conversely, low-SDI areas, with their low level of medical care and large cigarette populations, might result in more TB deaths; an estimated 80% of the world’s 1.1 billion smokers live in low- and middle-income countries, which had the highest cigarette smoking-related disease and death. Furthermore, the highest wave of TBC ASDR was in the 40–60 years old with 1920−40 birth cohort, indicating that the middle-aged cohort of TB patients who smoke was more prevalent than the older cohort, which was more different from TBA.

Based on the the cause of death composition analysis, TBA deaths were than those of TBC in 1990 globally, yet this situation reversed in 2019. In terms of SDI, both high and low SDI regions had more TBA than TBC, while by 2019, all SDI regions excepted low and medium SDI regions had higher TBA- than TBC-related deaths. This shift suggests that TBA changed over time and increased as a proportion of total TB deaths by 2019, suggesting TBA needed more attention.

Based on previous literature, the relationship between alcohol consumption and TB could be explained by four causal pathways. Biologically, consumption impairs the immune system and increases susceptibility to TB infection, as it affects ability of alveolar macrophages to respond to newly introduced pathogens. Since both alcohol and Mtb could increases pulmonary oxidative stress, alcohol's inhibition of nuclear factor erythroid 2-related factor 2 and the depletion of glutathione stores could potentiate Mtb infection and growth, while also disrupting immune function [29]. The oxidised alveoli in people who use alcohol provide a more favorable environment for Mtb, as it grows and survives more easily in oxidative environments [30]. From a pathological standpoint, prolonged and heavy exposure to alcohol impairs mucociliary clearance and predisposes to bronchodilation and aspiration [31]. Meanwhile, from a social behavior standpoint, alcoholics might be more likely to socialise with other alcohol drinkers who had a higher incidence of TB and were more likely to have smear-positive TB, increasing the risk of TB infection [32]. From a disease risk view point, alcohol consumption was associated with malnutrition, liver disease, and social drift, which promoted the risk of TB infection and spread of Mtb [33,34]. Since each nation had its own historical dynamics and trajectory, there might be heterogeneity of the association between TB and alcohol, more international researches should be conducted for the mechanism between TB and alcohol.

To reduce the impact of alcohol consumption on TB, governments could implement interventions to reduce the harms of alcohol consumption, including by regulating the environment in which alcohol is sold, its price and availability, but also by delivering interventions that individually target the risk level of alcohol consumption [35]. While cigarette smoking was associated with an increased risk of TB, this susceptibility might be due to increased exposure to Mtb, suppressed anti-TB immunity, and enhanced immunosuppressive N2 neutrophil or Treg activity in smokers due to increased cough [36,37]. To further achieve the goal of TB control and elimination, stakeholders should raise awareness of cigarette smoking-related health risks among TB patients and health care providers, while established TB treatment systems should routinely offer cigarette-dependent treatment in smoke-free health care settings.

Although the results of the GBD 2019 reflected the burden of TB prior to the emergence of the COVID-19 pandemic, the pandemic itself significantly affected the global TB burden in ways that still need to be explored through initiatives such as the Tuberculosis Program. Our ML algorithm predicted that both TBA and TBC ASDR would decrease in the future, but would not achieve the global goal of 95% reduction in mortality until 2035 [38]. In contrast to our results, a simulation study suggests that TB deaths might increase by 20% in the next five years [39,40]. Regardless of the rate of future TB deaths, policies of maintaining physical distance and wearing masks might help reduce TB transmission, yet they still might be offset by increased opportunities for household transmission. If TB treatment was interrupted because of health system overload due to COVID-19, the duration of infectiousness might have been further prolonged. Consequently, the Tuberculosis Program might need to consider recalibrating global targets once it had assessed the impact of COVID-19 on TB.

This study has several limitations. First, the assessment of TB mortality in the GBD database for countries without vital registration data was reported from verbal autopsies and verbal autopsy studies, which were generally sensitive in determining TB deaths. Second, this is an ecological study, allowing us to analyse the association between sex, age, SDI, and TB ASDR, yet preventing us from determining causal inference between risk factors and TB ASDR, which requires prospective studies. Furthermore, our interpretation is applicable at the population level, but might not be suitable for individuals. Third, we constructed an APC model a five-year multiplier period since GBD provided data at five-year intervals, which might smooth out some of the subtle changes in age, period, and cohort effects. Fourthly, there might be superior algorithms beyond ML models for predicting TB ASDR, and we only four in this study.

## CONCLUSIONS

ASDR for TBA declined worldwide, especially in Andean Latin America, East Asia, and Central Latin America, yet increased in a small number of regions, particularly in Central Asia. TBC declined more rapidly than TBA in several regions, particularly East Asia, North Africa and Middle East, and Andean Latin America, but increased only in part of Eastern Europe. Both TBA and TBC ASDR were much higher in men than in women; regarding age, TBA was highest in older adults aged 60–80 years, while TBC was highest in middle-aged adults aged 40−60 years. However, the rate of decline of TBA and TBC in both groups, combined with the XGBoost prediction results, showed that the ASDR for TBA was 1.29 (per 100 000 population) and the ASDR for TBC was 0.37 (per 100 000 population), suggesting that we might not achieve the 2035 global TB control target. Therefore, strengthening cigarette and alcohol control, especially for older men who drink alcohol, remains important for TB control.

## Additional material


Online Supplementary Document
