# Supplementary material for: Global, regional, and national mortality of tuberculosis attributable to alcohol and tobacco from 1990 to 2019: A modelling study based on the Global Burden of Disease study 2019
Source: J Glob Health. 2024 Jan 5;14:04023. doi: 10.7189/jogh.14.04023 (PMC10767425; doi:10.7189/jogh.14.04023)
Supplement: Online Supplementary Document [file jogh-14-04023-s001.pdf]

Online Supplementary Document

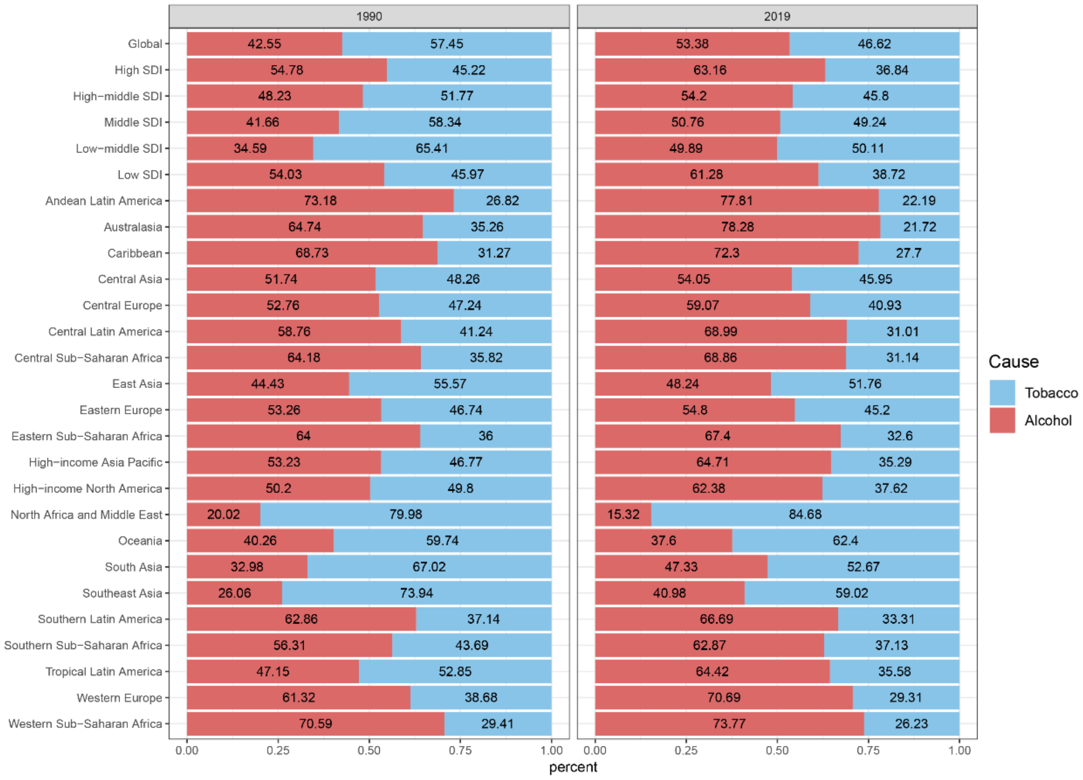

Figure S1 Composition chart of TB deaths related with alcohol and tobacco in 1990 and 2019.

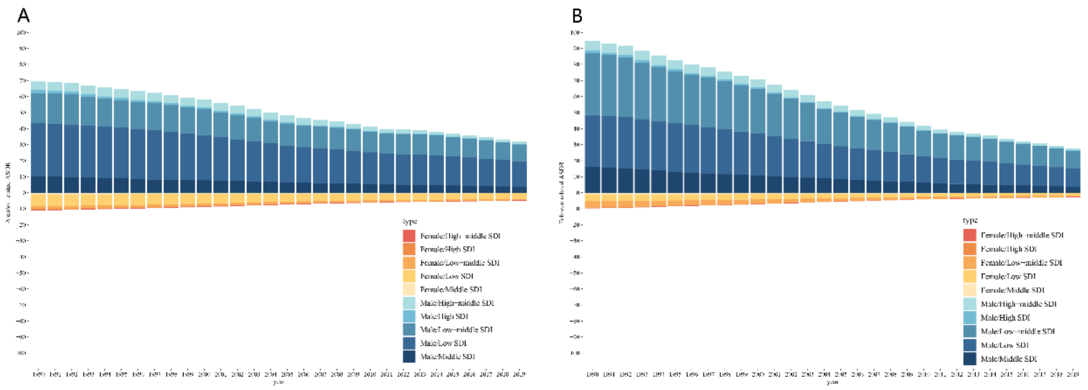

Figure S2 A: Alcohol-related ASDR distribution in different SDI area and gender; B: Tobacco- related ASDR distribution in different SDI area and gender.

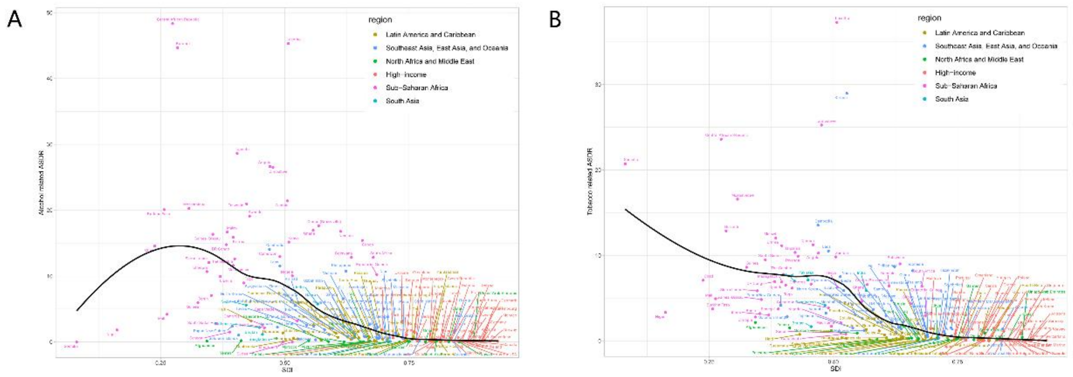

Figure S3 A: Alcohol-related correlation between SDI and ASDR in country level; B: Tobacco-related correlation between SDI and ASDR in country level.

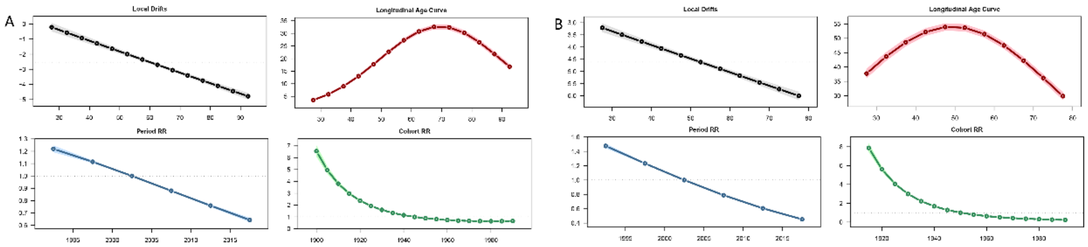

Figure S4 A: Alcohol-related ASDR APC model; B: Tobacco- related ASDR APC model.

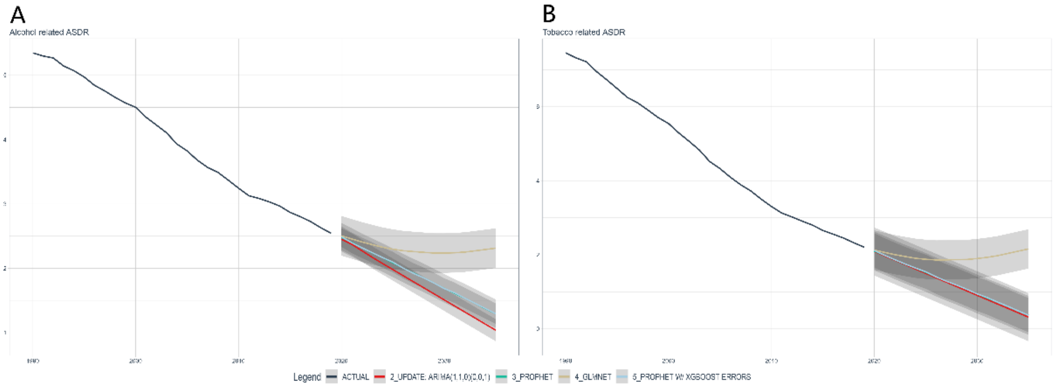

Figure S5 A: Machine learning prediction in alcohol related ASDR; B: Machine learning prediction in tobacco related YLLs.
